# Drugsniffer: An Open Source Workflow for Virtually Screening Billions of Molecules for Binding Affinity to Protein Targets

**DOI:** 10.3389/fphar.2022.874746

**Published:** 2022-04-26

**Authors:** Vishwesh Venkatraman, Thomas H. Colligan, George T. Lesica, Daniel R. Olson, Jeremiah Gaiser, Conner J. Copeland, Travis J. Wheeler, Amitava Roy

**Affiliations:** ^1^ Department of Chemistry, Norwegian University of Science and Technology, Trondheim, Norway; ^2^ Department of Computer Science, University of Montana, Missoula, MT, United States; ^3^ Rocky Mountain Laboratories, Bioinformatics and Computational Biosciences Branch, Office of Cyber Infrastructure and Computational Biology, National Institute of Allergy and Infectious Diseases, National Institutes of Health, Hamilton, MT, United States

**Keywords:** virtual screeening, machine learning, computer aided drug design, de novo design, SARS-C0V-2, protein-ligand docking

## Abstract

The SARS-CoV2 pandemic has highlighted the importance of efficient and effective methods for identification of therapeutic drugs, and in particular has laid bare the need for methods that allow exploration of the full diversity of synthesizable small molecules. While classical high-throughput screening methods may consider up to millions of molecules, virtual screening methods hold the promise of enabling appraisal of billions of candidate molecules, thus expanding the search space while concurrently reducing costs and speeding discovery. Here, we describe a new screening pipeline, called *drugsniffer*, that is capable of rapidly exploring drug candidates from a library of billions of molecules, and is designed to support distributed computation on cluster and cloud resources. As an example of performance, our pipeline required ∼40,000 total compute hours to screen for potential drugs targeting three SARS-CoV2 proteins among a library of ∼3.7 billion candidate molecules.

## 1 Introduction

The war against viruses is largely fought using vaccines and therapeutic drugs. As of December 2021, there are 55 FDA-approved vaccines against 19 human viruses (FDA, 2021), while only three viruses are targeted by approved antiviral drugs (FDA, 2020b). This disparity is particularly visible in the context of the ongoing SARS-CoV2 pandemic, in which vaccines were produced at a remarkable speed and with excellent effectiveness (FDA, 2020a; Wouters et al., 2021), while effective antiviral agents (Mahase, 2021; Jayk Bernal et al., 2022) only arrived 2 years into the pandemic, and with very limited availability. Despite vaccine success, there remains a vital need for development of effective antiviral drugs due to a combination of vaccine hesitancy, incomplete vaccine availability, breakthrough infection risk, and the continued emergence of viral variants (Kaplan and Milstein, 2021). Beyond SARS-CoV2, the cost and limited exploratory scope of current drug discovery pipelines will hamper efforts to quickly respond to future pandemic needs, and are an obstacle to development of antiviral drugs for viruses primarily afflicting relatively poor populations (Adamson et al., 2021).

Modern drug development efforts rely on high-throughput screening (HTS) analysis, which involves automated physical evaluation of activity across a library of thousands to millions of candidate small-molecule drugs (Berdigaliyev and Aljofan, 2020). HTS can be complemented by computer-aided drug design (CADD) and virtual screening (VS), in which interactions between small-molecules and a targets are estimated using computational models. In particular, computational analysis holds the promise of enabling expansion of the number of considered molecules from millions to billions.

VS strategies are traditionally divided into two categories: ligand-based (LBVS) and structure-based (SBVS) methods. In LBVS methods, a known active ligand is used as the basis for a search for chemically and structurally similar molecules (Ripphausen et al., 2011), with no consideration of the target protein. In SBVS approaches, small molecules are computationally docked into target binding sites to estimate their activities (Maia et al., 2020); this approach depends on availability of structural information, and is computationally intensive. The two methods can be integrated either by combining results (Wilson and Lill, 2011; Wang et al., 2020), or by using LBVS methods to quickly establish a set of candidates subjected to subsequent SBVS docking analysis (Drwal and Griffith, 2013).

Table 1 provides a list of various open access VS tools. For large scale virtual screening of compound libraries, software pipelines such as VSpipe Álvarez-Carretero et al. (2018), VirtualFlow Gorgulla et al. (2020, 2021), AMIDEDarme et al. (2021) have been used. Many of these approaches make use of SBVS and facilitate the use of a variety of docking Bender et al. (2021) programs with significant emphasis on scaling the calculations. Recent GPU acceleration of docking (Santos-Martins et al., 2021) has improved throughput, but resource requirements are still exceedingly high. For example, an effort to performing one billion docking assays was reported to require 664K GPU hours and 4.64M core hours for a single VS analysis (Acharya et al., 2020). With the aim of automating hit-selection protocols and minimizing human intervention, artificial intelligence-driven VS. pipeline have also been introduced Gentile et al. (2020), Gentile et al. (2021); Yaacoub et al. (2021).

**TABLE 1:** Several open access software tools for virtual screening. In a number of the tools, such as dockECR and VirtualFlow, multiple docking programs are used to predict scores between a single target or multiple targets (merging and shrinking approach) and a library of compounds. The AMIDE software carries out large-scale chemical ligand docking over a large dataset of proteins with the aim of identifying potential side effects of new drugs. iDrug, Pharmit (for structure-based pharmacophore modeling), iStar, e-LEA3D, USR-VS (3D shape-based similarity), MTiOpenScreen and ChemicalToolbox are web-based platforms for computer-aided drug design. ChemicalToolbox allows for integration with other tools and workflows (molecular dynamics) that are part of the Galaxy software framework (https://galaxyproject.org/). e-LEA3D uses a *de novo* drug design strategy in which fragments or combination of fragments that fit a QSAR model or the binding site of a protein are identified. * iDrug uses a pocket structure to define the pharmacophore descriptors needed for LBVS. However, they do not explicitly calculate the interaction between a ligand and the pocket, such as docking. In our opinion, they are marginally SBVS.

Software	LBVS	SBVS	ADMET
dockECR Ochoa et al. (2021)	✗	*✓*	✗
MolAr Maia et al. (2020)	✗	*✓*	✗
iDrug Wang et al. (2014)	*✓*	✓*	✗
ChemicalToolbox Bray et al. (2020)	✗	*✓*	*✓*
VirtualFlow Gorgulla et al. (2020), Gorgulla et al. (2021)	✗	*✓*	*✓*
AMIDE Darme et al. (2021)	✗	*✓*	✗
VSPipe Álvarez-Carretero et al. (2018)	✗	*✓*	✗
DockBlaster Irwin et al. (2009)	✗	*✓*	✗
e-LEA3D Douguet (2010)	✗	*✓*	✗
Pharmit Sunseri and Koes (2016)	*✓*	✗	✗
iStar Li et al. (2014)	✗	*✓*	✗
USR-VS Li et al. (2016)	*✓*	✗	✗
MTiOpenScreen Labbé et al. (2015)	✗	*✓*	✗
DrugSniffer	*✓*	*✓*	*✓*

Herein, we describe our development and release of an open source, massively-scalable LBVS-filtered SBVS pipeline, called *drugsniffer*, that is designed to achieve the goal of virtually screening bioactive drugs from datasets of billions of probably-synthesizable small molecules in a much-reduced time budget. *Drugsniffer* is easy to install and manages the distribution of computation across cluster or cloud resources. It reduces the computational burden to 10s of thousands of compute hours for search across a library of billions of candidate molecules, and provides a framework in which future methodological advances can be incorporated and evaluated. Using an early iteration of *drugsniffer*, we assessed ∼3.7B molecules for binding potential against 3 SARS-CoV2 proteins (22 binding pockets), with total computational investment of ∼40 K compute hours. The results of our analysis were accepted as a finalist in Joint European Disruptive Initiative (JEDI) “billion molecules against COVID19” challenge (Le et al., 2021).

## 2 Methods

**FIGURE 1:**
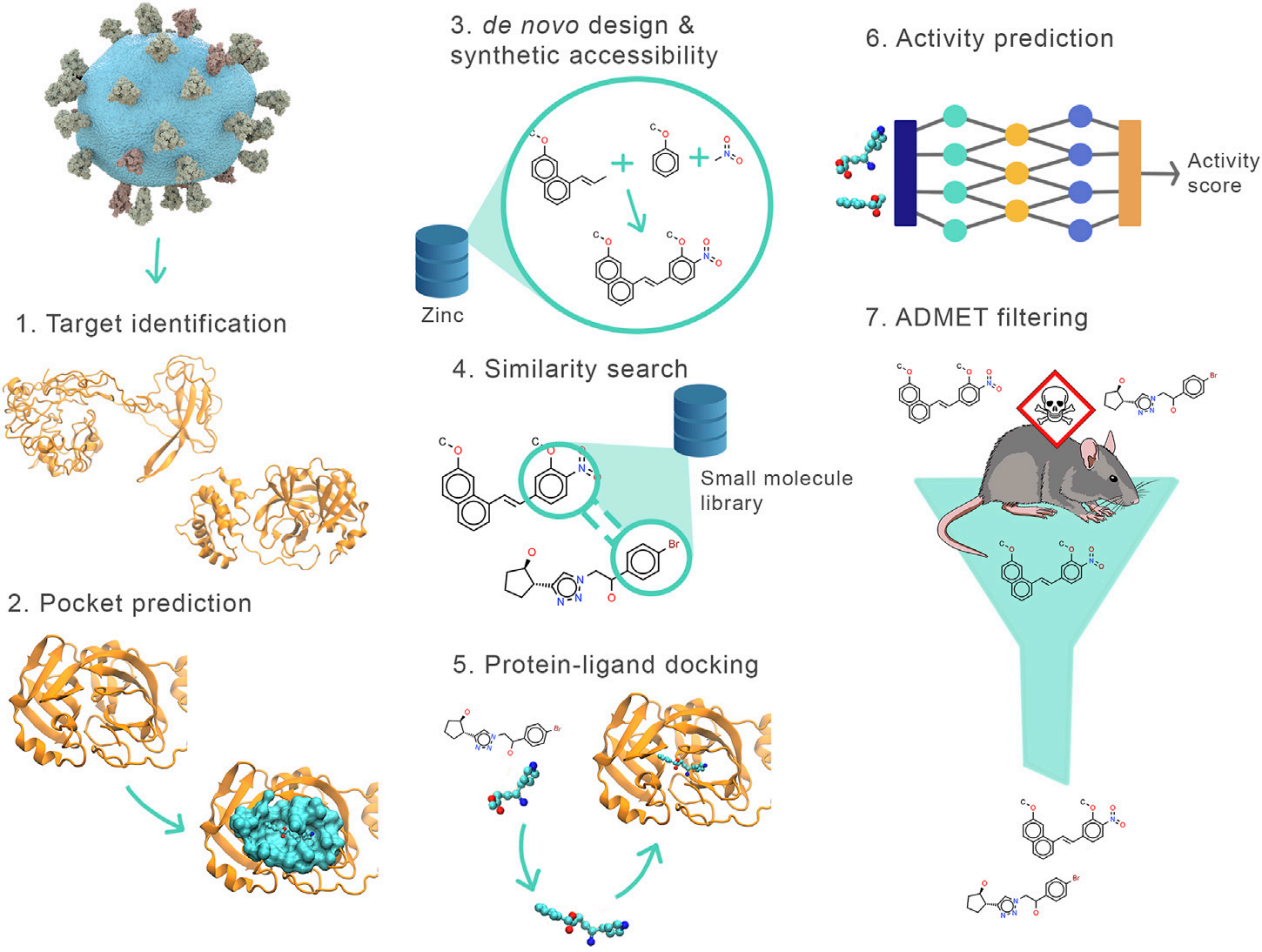
Outline of the drugsniffer virtual screening pipeline. The stages include (1) model the targets (e.g., using AlphaFold or crystal structure where available), (2) identify possible binding sites/pockets (e.g., using FPocket), (3) design multiple de novo ligands for the target pockets using AutoGrow, (4) use the designed molecules as seeds to identify similar compounds from small-molecule libraries (using ECFP4 fingerprints as found in RDKit), (5) dock the molecules (using AutoDock Vina) identified by the similarity search and calculate the interaction energy between the target and the docked poses, (6) re-score the best-docked poses of all the molecules using our new scoring function (terms for the function are provided by SMINA and DLIGAND2), (7) identify potentially toxic compounds using our fast ADMET analyzer (using FP-ADMET).

*Drugsniffer* consists of the following phases (see Figure 1): 1) select the protein target and determine its structure, 2) identify binding pockets, 3) design *de novo* ligands for each pocket, 4) use these as seeds to identify similar molecules in a large composite database of synthesizable small molecules, 5) perform *in silico* docking assays on these candidates, 6) apply a new neural network model to predict and rank binding affinity based on features of the docked poses, 7) identify potential toxicity of compounds using a custom ADMET filter. In this section, we describe these stages in detail, then discuss our application of an early implementation of the pipeline to the JEDI COVID19 Grand Challenge.

### 2.1 Selecting Target Proteins and Determining Structure

The first step in the drug screening process is the selection of the target protein–the user must provide a structural model for the selected protein. *Drugsniffer* is agnostic about the source of the structural model, and will work with experimentally-validated or computationally-predicted structures. Though protein structures may be retrieved from a variety of sources, we have had good experiences with ChimeraX (Pettersen et al., 2021), which, for example, supports retrieval of structures from the Protein Data Bank (Berman et al., 2000)) or prediction using AlphaFold2 (Jumper et al., 2021). AlphaFold2 achieved remarkable accuracy in the CASP14 competition; for example, in 92.5% of predictions, all side chain atoms are predicted with error ≤ 5 Å (Pereira et al., 2021). This accuracy is unprecedented for computational models, and these models may provide insight into the diversity of conformations that extend beyond the single conformer of a crystal-based structure. Even so, a substantial fraction of the predicted atoms, primarily from the flexible parts of the proteins, may not be modeled correctly by AlphaFold2. We encourage users to evaluate the overall (IDDT) and residue-specific (pLDDT) scores to evaluate the predicted accuracy of the overall and pocket regions of an AlphaFold2 model.

### 2.2 Identifying Pockets

In addition to a target protein structure, *drugsniffer* must be provided with at least one pocket descriptor, as well as a preferred pocket box size. The most reliable way of detecting a ligand-binding pocket is a user’s prior knowledge about the binding pocket from experience, experimental evidence, and literature search. Computational identification of a pocket-like region is challenging and an active area of research (Zhao et al., 2020). The *drugsniffer* pipeline includes a copy of the cavity detection software Fpocket (Le Guilloux et al., 2009) only because it is a stand-alone free program. We encourage users to use multiple pocket search algorithms, such as FTMAP Kozakov et al. (2015), POCASA Yu et al. (2010), and molecular dynamics simulations, and use their judgment to define a pocket-like region in the protein. The current implementation of the *drugsniffer* pipeline produces an FPOCKET output that includes all predicted pockets; the user is tasked with manually reviewing these and identifying the subset for which the downstream drug discovery stages should be performed, e.g., using ChimeraX or PyMol (Oliveira et al., 2014). Pocket descriptors identified outside of the *drugsniffer* pipeline may be provided as an alternative or supplementary source of predicted pockets. Box size must be determined for each pocket; we recommend basing this on the scheme proposed by (Feinstein and Brylinski, 2015).

### 2.3 *De Novo* Ligand Design

Following manual pocket identification, *drugsniffer* accepts as input the set of targeted pockets, and proceeds in an automatic fashion through the remaining stages. In the first stage, a large number of candidate ligand molecules are designed from scratch using the software AutoGrow4 (Spiegel and Durrant, 2020), which employs a genetic algorithm to evolve ligands from building blocks obtained from the ZINC library (Sterling and Irwin, 2015). AutoGrow4 utilizes a diversity score that acts as a secondary fitness metric and is used to select seed compounds that are structurally unique from previous generations. The molecules are subsequently docked into the pockets of the specified target protein using QuickVina (Alhossary et al., 2015) which is a faster version of Autodock Vina. Docked results are ranked based on the Vina docking score of the top ranking pose. A Lipinski RO5 filter is used to exclude candidate structures that do not satisfy drug-like criteria. The NIH filter (Jadhav et al., 2010) is also included to screen against compounds containing undesirable functional groups. AutoGrow4 performs *in silico* chemical reactions (Durrant and McCammon, 2012) derived from a set of robust organic reactions (Hartenfeller et al., 2011) to generate new child compounds from a parent molecule. These reaction-based structural transformations are used to increase the likelihood of the designed molecules being synthetically accessible. However, a drawback of using pre-defined reaction schemes is that they may match reaction handles and fail to consider the presence of competing functionalities that can compromise the reaction outcome (Ghiandoni et al., 2020; Meyers et al., 2021). By default, the pipeline runs AutoGrow4 for 10 generations, and captures 150 *de novo* molecules from each of the final three generations. *Drugsniffer* can optionally forgo this AutoGrow4 step, and instead accept a collection of ligands provided by the user–these may be sourced from some prior *de novo* computation, or from a collection of co-crystallized protein-ligand complexes.

### 2.4 Molecular Similarity Search

The motivation for employing *de novo* ligand design is to produce drug-like compounds that can mimic known inhibitors or potentially active ligands with a diversity of chemical structures. While the molecules produced by AutoGrow4 are predicted to be synthesizable, factors such as establishing synthetic routes, material procurement, costs and time involved are difficult to predict. We therefore sought to build on the value of these designed molecules through an LBVS search strategy in which the *de novo* molecules serve as seeds in a search for similar compounds within a massive library of molecules.

We compiled a collection of molecules from various small-molecule libraries, with the aim of capturing a large diversity of molecules that either already exist, or are likely-synthesizable and can be made to order (see Table 2). The Enamine collection includes more than 1 billion compounds that comply with Lipinski’s rule of five (RO5) criteria and are expected to be realized in 1–3 synthesis steps. The Synthetically Accessible Virtual Inventory (SAVI) (Patel et al., 2020) contains over 1 billion reliably-synthesizable compounds generated through expert-system rules. GDB-13 (Blum and Reymond, 2009) also contains over 1 billion compounds (containing up to 13 atoms of C, N, O, S, and Cl = , generated according to chemical stability and synthetic feasibility rules. PubChem (Kim et al., 2020), ZINC (Sterling and Irwin, 2015), and Molport are curated collections of commercially-available molecules. SweetLead (Novick et al., 2013) and DrugBank (Wishart et al., 2017) contain drugs that are in use or in clinical trials, and may therefore facilitate repurposing of established drugs. We removed molecules containing salts, because downstream docking methods fail in the face the apparent disjoint molecules. The full de-duplicated collection contains ∼3.7 billion unique molecules.

**TABLE 2:** The small molecule databases searched as part of the VS protocol.

Database	Number of ligands
Sweetlead	≈4,000
Drugbank	≈10,000
MOLPROT	≈7,600,000
PUBCHEM	≈103,000,000
ZINC15	≈417,000,000
GDB	≈1,003,000,000
SAVI	≈1,009,000,000
ENAMINE	≈1,200,000,000
Total	≈3,700,000,000

https://simtk.org/projects/sweetlead

https://www.drugbank.ca/releases/latest

https://www.molport.com/shop/libraries-collections

http://ftp.ncbi.nlm.nih.gov/pubchem/Compound/

http://files.docking.org/catalogs/

http://gdb.unibe.ch/downloads/

https://cactus.nci.nih.gov/download/savi_download/

https://enamine.net/library-synthesis/real-compounds/real-database

To identify library-sourced compounds similar to the *de novo* seeds produced by AutoGrow4, 1024-bit ECFP4 fingerprints (O’Boyle and Sayle, 2016) are computed for all ∼3.7 billion library compounds. The ECFP4 fingerpint is a 1024-element binary vector that encodes structural and chemical features. Though a multitude of fingerprint strategies exist, ECFP4 has been reported to effectively rank diverse structures by similarity (O’Boyle and Sayle, 2016). Future releases of *drugsniffer* will enable selection of other fingerprints, or related similarity measures. ECFP4 fingerprints are computed using RDKIT (https://www.rdkit.org), then stored as a sequence of 1,024 bit vectors, so that a library of 3.7 billion molecules is represented by a ∼475 Gbyte fingerprint database. Fingerprints are similarly computed for all seeds. A measure of similarity between two molecules is computed by comparing the 1024-bit fingerprints of each molecule, using the Tanimoto coefficient (aka Jacaard index): the ratio of the intersecting set (number of bits set to one in both fingerprints) to the union set (number of bits set to one in at least one of the two fingerprints) (Bajusz et al., 2015). Similar (“neighbor”) molecules are identified by computing the Tanimoto coefficient for each seed against each molecule in the fingerprint database using SIMD vectorized bit-level comparison over 1,024 representative bits per molecule. By default, neighbors with Tanimoto similarity > 0.5 to at least one seed are captured for later docking estimates. This threshold is selected based on experience, with the aim of balancing stringency (reducing the computational burden of later stages) with permissiveness (expanding the pool of candidates that reach the next stage); it can be altered at run time.

### 2.5 Protein-Ligand Docking

For the seed-neighbor molecules identified by the similarity search, initial 3D coordinates are generated from the SMILES representations using OpenBabel (O’Boyle et al., 2011a). Diverse low-energy conformers for the molecules are generated using the Confab (O’Boyle et al., 2011b), then the lowest energy conformation is retained. These optimized structures of neighbors are docked into their respective targets using AutoDock Vina (Trott and Olson, 2010). The number of docking poses produced and the exhaustiveness parameter for the search for each ligand are parameterized by the user; the default values are 9 and 4, respectively.

### 2.6 Re-Scoring Docked Ligands, to Estimate Binding Affinity

AutoDock Vina reports a set of molecular poses within the pocket, along with a value representing a prediction of the quality of each docked pose. Because this prediction is only a loose estimate of actual binding affinity, a variety of post hoc re-scoring methods have been devised [e.g., see (Koes et al., 2013a; Chen et al., 2019; McNutt et al., 2021)]. *Drugsniffer* can report either the Autodock Vina score, the SMINA (Koes et al., 2013b) rescoring value, or the result of a new neural network re-scoring strategy that we have produced for this workflow (*dock2bind*, which is the default). *Drugsniffer* supports retraining of this model with domain-specific binding affinity data, and also will accept an alternate re-scoring function that is injected by the user into the *drugsniffer* wokflow by providing a Docker container meeting a simple documented API.

**FIGURE 2:**
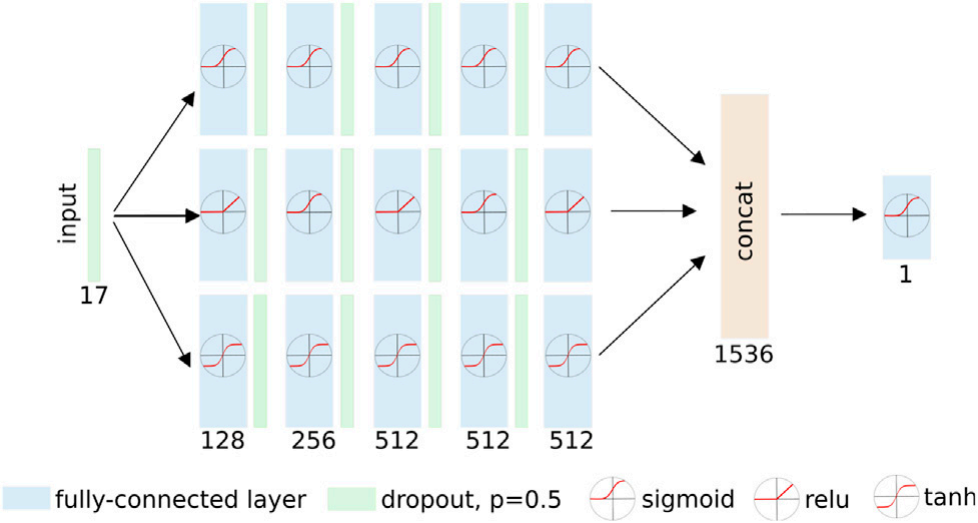
Affinity prediction model. The model consists of three separate paths from input to output, each composed of five sequential fully-connected layers. Each path uses a separate set of activation functions, allowing the network to learn diverse representations of the input. The outputs of the three paths are concatenated and passed through a final fully-connected (FC) layer that emits a prediction of binding or non-binding. Fully-connected (FC) layers are represented with blue blocks. The number of nodes in each FC layer is indicated below the block. Activation functions applied to the output of the FC layers are shown in circles. The model was trained for 2000 epochs and batch size 8,192 with the Adam (Diederik and Ba, 2014) optimizer using default *β*_1,2_ parameters and a learning rate of 0.001. Dropout (Srivastava et al., 2014) with *p* = 0.5 was applied after each fully connected layer during training, and also during validation.

For each docked pose, our *dock2bind* receives 16 pose descriptors calculated by SMINA, along with the DFIRE estimate of protein–ligand potential (Chen et al., 2019), and computes a new affinity estimate for the pose. This estimate is a value between 0 and 1 and can be thought of as the model’s confidence that the molecule binds to the pocket, constrained by the specific pose. See Figure 2 for model details. Ligand-protein pairs were taken from the DUD-E benchmark (Mysinger et al., 2012) and LIT-PCBA (Tran-Nguyen et al., 2020). To train the model, docked poses were generated for ∼14,000 ligand-protein pairs from the DUD-E dataset, along with ∼800,000 decoy ZINC-sourced compounds docked to the same protein partners. These were supplemented with an additional ∼4,000 ligand-protein complexes from LIT-PCBA, and ∼121,000 decoys docked to the same proteins. The active:decoy ratio is intended to reflect the large actual classification imbalance (most molecules are inactive for any specific target). For each target, 9 docked poses were produced, and the pose with the best SMINA score was provided to the *dock2bind* model for training.

### 2.7 ADMET Analysis

*Drugsniffer* includes a suite of models to predict properties tied to bioavailability and safety. Owing to their ease of computation, molecular fingerprints have been frequently used to predict these properties (Kim and Nam, 2017; Ai et al., 2018; Yang et al., 2019). Fingerprint-based classification models were trained on experimental data available [see (Venkatraman, 2021)] for solubility in dimethyl sulfoxide (DMSO), blood brain barrier permeability, human intestinal absorption (HIA), AMES mutagenicity, HERG cardiotoxicity, drug induced liver injury (DILI), Cytochrome p450 interaction (CYP3A4 and CYP2C9 isoforms), metabolic stability and acute LD_50_ toxicity based on the criteria defined by the Environmetal Protection Agency (EPA). For each property, various fingerprints (Hinselmann et al., 2011) (substructure and extended/functional connectivity fingerprints) were evaluated for their discriminant ability and the fingerprint model [using random forests (Breiman, 2001)] yielding the best balanced accuracy (Brodersen et al., 2010; Venkatraman, 2021) was retained. The *drugsniffer* pipeline applies these models to the list of candidates produced by previous stages, and appends the resultant vector of properties to the affinity prediction results. The models can be accessed at https://gitlab.com/vishsoft/fpadmet.

### 2.8 Software and Data

*Drugsniffer* is implemented as a Nextflow workflow (Di Tommaso et al., 2017) that orchestrates the activity of a curated set of open source tools, and supports analysis in cluster (SLURM) and cloud (AWS) environments. Table 3 lists the different software tools that are used in the workflow. The workflow depends on a collection of Docker containers and runner scripts wrapping each of our own tools as well as the external open source tools included in the analysis pipeline. This organizing principle makes it possible for the user to configure and run *drugsniffer* without concern for dependencies. Docker container files, NextFlow scripts, and tool code are all available *via* GitHub (https://github.com/TravisWheelerLab/drug-sniffer). Versioned Docker container images are published in the GitHub containiner registry, and the full library of ∼3.7 billion molecules (with pre-computed fingerprints) is housed in a persistent OSF repository (Soderberg, 2018) and. Instructions for download and use are found at http://drugsniffer.org.

**TABLE 3:** Software used in the VS pipeline.

Software	Comments
RDKit	Routines for ECFP4 fingerprint generation
Chemistry Development Kit	logP estimation routines
OpenBabel	interconvert chemical file formats
MGLTools	interconvert chemical file formats
AutoDock Vina	Protein-ligand docking
DLigand2	statistical potential term for protein-ligand binding affinity prediction
SMINA	scoring terms for protein-ligand binding affinity prediction
AUTOGROW4	*de novo* ligand design using docking
FP-ADMET	Prediction of ADMET properties

https://www.rdkit.org

https://cdk.github.io/

http://openbabel.org/wiki/Main_Page

https://ccsb.scripps.edu/mgltools/downloads/

https://github.com/ccsb-scripps/AutoDock-Vina

https://github.com/sysu-yanglab/DLIGAND2

https://github.com/mwojcikowski/smina

https://git.durrantlab.pitt.edu/jdurrant/autogrow4

https://gitlab.com/vishsoft/fpadmet

### 2.9 Application of *Drugsniffer* to JEDI COVID19 Grand Challenge

In May 2020, the Joint European Disruptive Initiative (JEDI) launched a “Grand Challenge” competition intended to motivate development of methods capable of searching a library of billions of molecules for those with potentially good binding affinity for target SARS CoV2 proteins. We developed *drugsniffer* to meet these goals, and submitted candidate molecules identified with an early version of the piepeline. Our submissions have reached the finalist stage, and are currently under experimental review. Here, we describe how our pipeline was used to prepare our submission, and document the differences between the version of the pipeline used for our JEDI submission and its current released form.

To begin, we selected three target proteins: RNA dependent RNA polymerase (Non-structural Protein 12, aka NSP12), 3C like protease (3CLPro), and Nucleocapsid protein (N). At the time of the analysis, no whole-protein experimental structure was available for any of the targets and AlphaFold2 was not yet released. We therefore downloaded models created by I-TASSER (Yang et al., 2015), and added hydrogen atoms with CHARMM (Brooks et al., 2009).

Candidate binding pockets for the three selected targets were identified using a combination of literature search and results from the tools FTMAP (Kozakov et al., 2015) and POCASA (Yu et al., 2010) (*drugsniffer* incorporates Fpocket in lieu of these, because its license allows redistribution). Seven pocket-like regions were identified: 2 each for N and 3CLpro, and 3 for NSP12. Some of the pocket-like regions were too large to be occupied by a typical-sized ligand. Consequently, the larger pocket-like regions were subdivided into smaller pockets. A total of 22 pockets were finalized as targets: 8 each for N and NSP12 and 6 for 3CLPro. We searched the literature to identify any glycosylation sites for the three selected targets and did not find any. We also used N-GlyDe (Pitti et al., 2019) to identify any potential sites for N-linked glycans. Our predicted glycosylation sites are residue 269 of N and residues 767 and 911 of NSP12. As none of the glycosylation sites were near any of the predicted binding pockets, we did not consider glycosylation for our later docking exercises.

The next several pipeline stages were run as in the current release of the pipeline, including *de novo* ligand design, molecular similarity search, and protein-ligand docking. AutoGrow4 was run for 25 generations, over five independant runs. In total, 31,962 seed molecules were identified by AutoGrow4 (12,227 for nsp12 pockets, 14,334 for N pockets, and 5,401 for 3CLPro pockets). Molecular similarity search identified ∼97,000 library compounds with Tanimoto similarity > 0.6 to some seed, and another ∼955,000 with Tanimoto similarities of 0.5–0.6. Among the 97,000 closest neighbours: ∼43,000 were identified for nsp12, ∼34,000 for N, ∼20,000 for 3CLPro. For each pocket, all seed neighbor molecules were docked (AutoDock Vina) to the pocket, and poses were re-scored using dock2bind, using the top re-scored pose for each molecule as its predicted affinity. The top-scoring 30,000 candidates (10,000 per protein) were analyzed for ADMET and predicted synthetic complexity [SCSCORE (Coley et al., 2018)] of the target molecule. Candidates with no ADMET contraindications, and with an expected number of synthesis steps ≤5 were submitted to the JEDI challenge; 18 compounds passed JEDI criteria for the final evaluation, and are being synthesized and evaluated.

## 3 Results

Here, we have described the stages and availability of a new pipeline for exploring a pre-built library of billions of likely-synthesizable molecules for a small set of candidate molecules that are expected to show good binding affinity to a user-provided protein structure and pocket descriptor. As a proof of principle, we used a variant of this pipeline to identify drug candidates from our library of ∼3.7 billion molecules, targeting 22 pockets in 3 proteins associated with SARS-CoV2, resulting in a list of ∼30,000 candidate compounds. This collection was submitted for analysis to the JEDI “Grand Challenge,” and were advanced to “finalist” status; experimental review of a subset of these molecules is underway. Compute time for the total search for candidate molecules for all 22 pockets was ∼40,000 CPU hours. By distributing workload across a cluster, the analysis required only a few days. In addition to these run time results, we explored the efficacy of our custom docking re-scoring model, as well as the outcomes of ADMET and synthesizability analysis.

### 3.1 Performance of the Deep Learning Re-Scoring Model

**FIGURE 3:**
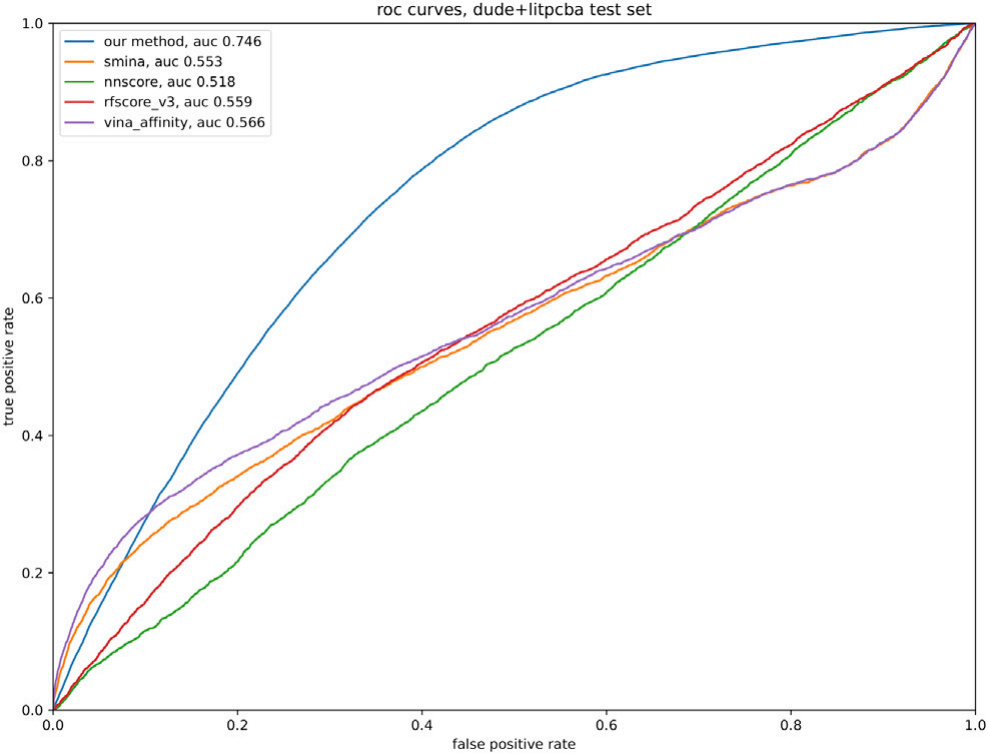
Test data consisting of 3,900 ligand-protein pairs and 213,000 decoy-protein pairs was analyzed with the tools listed in the legend, with the relevant tool producing a binding affinity estimate for each pair. Default parameters were used for all tools; our model was trained as described in the text. A ROC curve was produced for each tool, based on the sorted list of predicted affinity.

To quantitatively evaluate our model, a test set was developed from DUD-E and LIT-PCBA, consisting of complexes involving proteins not found in the training set. A total of ∼3000 DUD-E ligand-protein pairs, ∼186,000 decoys for DUD-E proteins, ∼900 LIT-PCBA ligand-protein pairs, and ∼27,000 decoys for LIT-PCBA. No hyperparameter tuning was performed on any of the models so a validation set was unnecessary. To test the efficacy of our method of ranking potential binders, we compared our method to a variety of open-source implementations of affinity-predicting methods, including Vina’s default method, the SMINA default score, and the NNScore and RF-score (version 3) from the Open Drug Discovery Toolkit (Wójcikowski et al., 2015) (ODDT). Figure 3 shows the performance of the model architecture trained on different subsets of the data.

### 3.2 ADMET and Synthesizability Analysis

**FIGURE 4:**
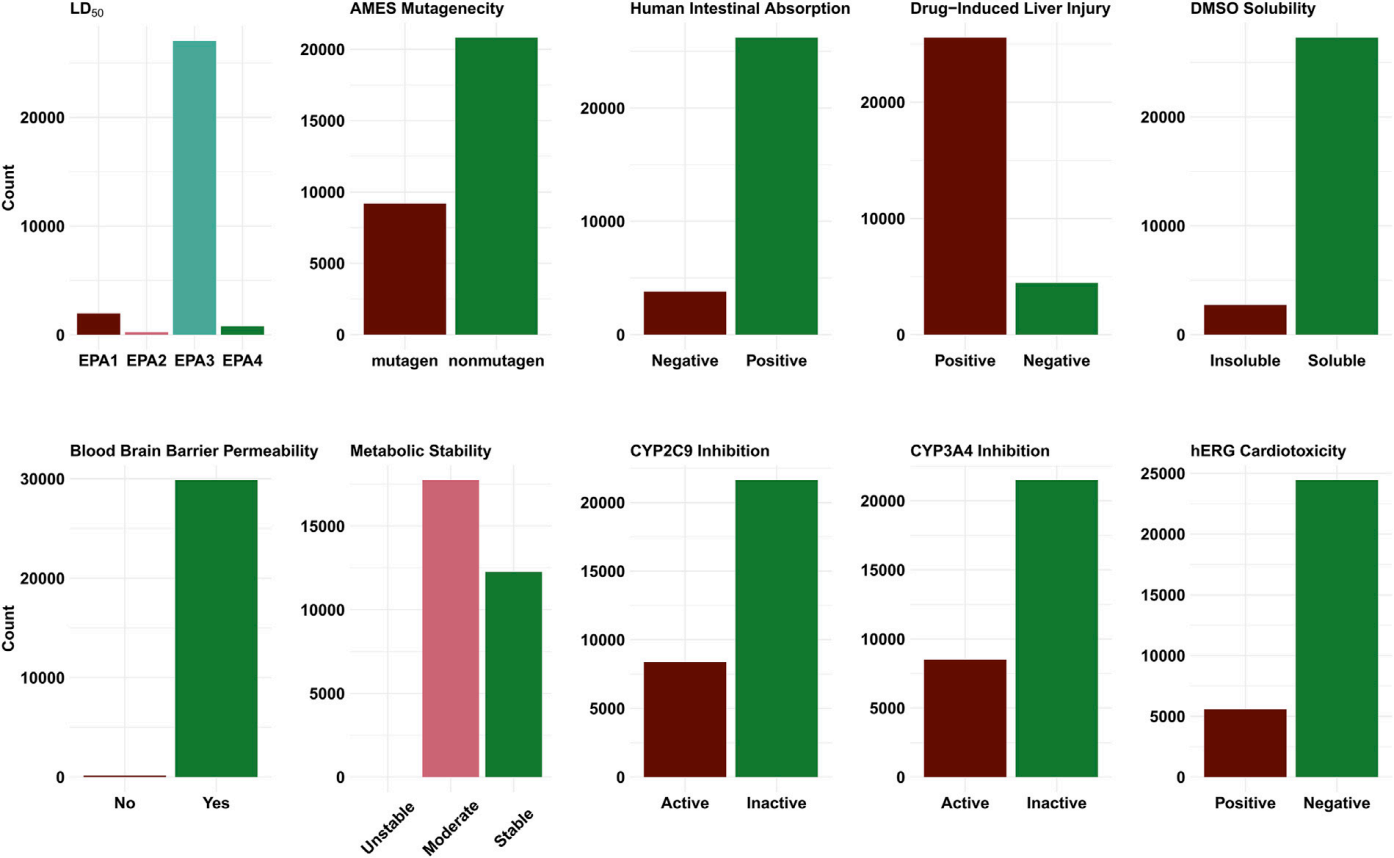
Bar plots showing the distribution of the predicted classes for the different ADMET endpoints. EPA1 corresponds to the (LD_50_ ≤ 50 mg/kg) the highest toxicity category. EPA2 (moderately toxic) includes chemicals with 50 < LD_50_ ≤ 500 mg/kg. EPA3 (slightly toxic) includes chemicals with 500 < LD_50_ ≤ 5,000 mg/kg. Safe chemicals (LD_50_ > 5,000 mg/kg) are included in EPA4. Here, the color green is used to indicate compounds that are better suited for further study.

Figure 4 shows the distribution of the ADMET properties for the ∼30,000 compounds that were submitted to the JEDI competition. For the most part, the shortlisted compounds were predicted to have favourable ADMET properties. Our ML model for DILI (Venkatraman, 2021) predicts a majority (∼85%) of the compounds to be hepatotoxic. The DILI model however only provides a binary (yes/no) prediction and does not indicate the level of the underlying DILI severity. A strict application of the models (i.e., selecting only those compounds that are deemed to be favourable across all calculated properties) yielded a set of 1,635 compounds. Many ADMET properties are affected by the dosage, route and frequency. For better assessment of ADMET, knowledge of the underlying mechanisms is required. Given that it is far from trivial to prioritize one property over the other (leading to varying application of the filter), we have used the model predictions as a guide rather than a filter. With respect to synthesizability, ∼79% of molecules identified by the pipeline were predicted to require three or fewer predicted reaction steps.

## 4 Discussion

Virtual screening has seen a recent rise in prominence, supported by improved computational methods across the range of analyses represented in the *drugsniffer* pipeline. The ongoing pandemic has highlighted the need for improved speed and increased exploratory scope of virtual screening methods. Relatedly, the development of low-cost virtual screening methods holds the promise of improving opportunities for development of drugs targeting diseases prevalent in low-income regions, for which economic incentives discourage expensive high-throughput screening assays. We developed *drugsniffer* as a preliminary tool to meet this need, exploring billions of candidate molecules for a target protein pocket in a few thousand compute hours–relatively modest resources available to most HPC infrastructures. Even with its development, each of the stages of the *drugsniffer* pipeline will be well-served by methodological advances. We highlight a few such areas of opportunity here, and observe that *drugsniffer* can easily adapt to incorporate advances along these lines, due to its modular nature.

With the development and release of AlphaFold2 and similar structure prediction methods, structure prediction is perhaps no longer a general bottleneck in the drug discovery problem, though some protein types still suffer from relatively uncertain predictions. Pocket identification remains a challenge, and most current techniques can detect pockets only with ∼60% accuracy (Zhao et al., 2020). Advances in this field will reduce the dependency on expert manual analysis of structures and pockets.

### 4.1 Future Advances

*Drugsniffer* will also be improved by development of advances in *de novo* molecule production (where limitations include wall clock run time and molecule synthesizability and utility), molecular similarity search (where current molecule-centric approaches fail to account for pocket-specific interaction profiles), and docking-based affinity prediction (where re-scoring methods produce only modestly enrichment for actives vs. decoys (see Figure 3) and may not generalize well to structures that are not represented in the training set). *Drugsniffer* will be expanded by including molecular dynamics simulations to consider multiple conformations of a pocket region and refining binding energy estimation of shortlisted ligands. It should be emphasized that the scope of the *drugsniffer* pipeline is to identify possible ligands with high enrichment factors. Users should carry out such MD or QM studies on the possible ligands predicted by the *drugsniffer* for a more accurate prediction of binding affinity or to investigate the effect of protonation states in binding. Due to their approximate nature, docking forcefields are insensitive to such details.

## Data Availability

Publicly available datasets were analyzed in this study. This data can be found here: http://drugsniffer.org.
